# Introduction of Routine Zinc Therapy for Children with Diarrhoea: Evaluation of Safety

**Published:** 2007-06

**Authors:** A.M. Khan, C.P. Larson, A.S.G. Faruque, U.R. Saha, A.B.M.M. Hoque, N.U. Alam, M.A. Salam

**Affiliations:** 1 Clinical Sciences Division; 2 Health Systems and Infectious Diseases Division, ICDDR,B, GPO Box 128, Dhaka 1000, Bangladesh; 3 Department of Pediatrics, Department of Epidemiology, Biostatistics and Occupational Health, McGill University, Montreal, Canada; 4 Progoti Samaj Kallyan Protisthan Clinic, Dhaka, Bangladesh

**Keywords:** Diarrhoea, Diarrhoea, Infantile, Evaluation studies, Safety, Zinc therapy, Bangladesh

## Abstract

On 8 May 2004, the World Health Organization (WHO) and the United Nations Children's Fund (UNICEF) recommended routine administration of zinc in the management of children, aged less than five years, with acute diarrhoea. In making the recommendation, WHO and UNICEF also suggested careful monitoring for adverse events associated with routine administration of zinc, particularly unusual or excess vomiting. The study assessed, in a phase IV trial, i.e. post-marketing surveillance of zinc, the occurrence of adverse events during the first hour after the administration of the first dose of zinc in children with acute or persistent diarrhoea. The study was conducted at the Dhaka Hospital of ICDDR,B and at an outpatient clinic operated by a local health NGO—Progoti Samaj Kallyan Protisthan (PSKP), Dhaka, Bangladesh. Eligible children, aged 3-59 months, were treated with 20 mg of zinc sulphate provided in a dispersible tablet formulation. The children were observed for 60 minutes following the initial treatment with zinc for adverse events, with particular attention given to vomiting or regurgitation. During the one-year observation period, 42,440 children (male 57% and female 43%) received zinc, and 20,246 (47.8%) of them were observed. Regurgitation and/or vomiting occurred in 4,392 (21.8%) of the children; 90.8% of these children had vomiting only once, 8.7% twice, and 0.5% more than twice. No children revisited the hospital for recurrent vomiting following their discharge. A significant proportion of infants and children may experience vomiting or regurgitation, usually once, following the administration of the first dose of zinc. This is a transient phenomenon that did not impact on continuation of treatment with zinc.

## INTRODUCTION

Zinc, a micronutrient, is a component in many metallo-enzymes and poly-ribosomes involved in cellular function. It is essential for metabolism, cellular growth, and immune function in humans (1). Zinc deficiency is widespread among children in developing countries where serious infectious diseases, such as diarrhoea, are common (2–3). The therapeutic effects of zinc in acute and persistent diarrhoea have been investigated in several studies and have consistently benefited young children in terms of reducing the severity and duration of episodes of diarrhoea and subsequent prevention of episodes (4–14). The World Health Organization (WHO) has estimated that 750,000 children die globally each year due to zinc deficiency (15), and it is estimated that over half of these deaths could be prevented through routine treatment with zinc as part of the management of childhood diarrhoea (16). Based upon all the evidence and in response to the recommendations of WHO/United Nations Children's Fund (UNICEF) (17), ICDDR,B decided to introduce zinc therapy in the routine management of children with acute or persistent diarrhoea at its hospital in Dhaka, Bangladesh.

Side-effects, such as nausea and vomiting, following ingestion of zinc for the treatment of various medical problems have been reported in adults and adolescents, but at higher doses, for example, in the management of non-responsive coeliac disease, sickle cell anaemia, and apthous ulcer (18). Acute zinc toxicity due to excess administration (225-450 mg of zinc) includes gastrointestinal symptoms, such as nausea, vomiting, epigastric pain, abdominal cramps, and bloody diarrhoea (18). Recent randomized clinical trials in adults and adolescents with acne, anorexia nervosa, macular degeneration, common cold, and tuberculosis, which used elemental zinc in the dose range of 50-100 mg/day, reported nausea and vomiting as side-effects (19–22). In two clinical trials with zinc among children, aged less than five years (under-five children) with diarrhoea, vomiting was the only side-effect observed (10,23). In view of this information on zinc-related side-effects and the joint WHO/UNICEF recommendations to monitor for adverse events associated with zinc therapy, this study was undertaken to evaluate regurgitation and vomiting following the initial treatment with zinc.

## MATERIALS AND METHODS

### Study design and approval

This was a phase IV safety study, i.e. post-marketing surveillance of treatment with zinc for childhood diarrhoea to evaluate the occurrence of adverse events. The Research Review Committee and the Ethical Review Committee of ICDDR,B reviewed and approved the study.

### Study time and site

The study was conducted during April 2004–March 2005 at the Dhaka Hospital of ICDDR,B and an adjacent outpatient clinic operated by a local health NGO— Progoti Samaj Kallyan Protisthan (PSKP)—located on the Centre's premises. The Dhaka Hospital and the PSKP clinic provide treatment to around 110,000 patients with diarrhoea each year, most of whom are residents of urban and peri-urban Dhaka, the capital city of Bangladesh. The majority of the patients come from a poor socioeconomic background, living in city slums (Faruque ASG. Personal communication, 2006). Under-five children constitute about 50% of all patients, with malnutrition being very common among them. About 35-40% of all children attending the Dhaka Hospital are referred to and treated at the PSKP clinic, and about 55% are treated in the Short Stay Ward (SSW) of the Dhaka Hospital. The remaining 5-10% are either admitted to an intermediate care ward or transferred to the appropriate health facility.

### Clinical practice guideline for zinc and study administration

Children of either sex, aged 3-59 months, attending the Dhaka Hospital of ICDDRB with uncomplicated diarrhoea, either admitted to the SSW or referred to the PSKP clinic, constituted the source population. Those with co-morbidities associated with excessive vomiting, e.g. whooping cough, pylorospasm, congenital pyloric stenosis, hiatus hernia, other obstructive defects of the gastrointestinal tract, cow's milk allergy, etc., were excluded from routine zinc therapy. Similarly, in addition to diarrhoea, children with any other systemic complications, e.g. pneumonia, meningitis, sepsis, paralytic ileus, severe malnutrition, hypoglycaemia, serum electrolyte disorders, seizure, etc. that required admission to the longer-stay wards of the Dhaka Hospital, were also excluded. The decision of administering zinc, outside this study, was left to the respective care-giving doctors.

Children were initially triaged and those with uncomplicated, mild diarrhoea having no sign of dehydration were referred to the PSKP clinic. Children with signs of dehydration, but without serious co-morbidity, were admitted to the SSW of the Dhaka Hospital.

In the PSKP clinic, the usual stay of the children was 1-2 hour(s), where they were given oral rehydration salts solution (ORS) and their usual foods. Here, children who fulfilled the study criteria were given their first dose of zinc when they were considered settled, i.e. not dehydrated, no vomiting in the last 30 minutes, and taking ORS as instructed. A trained research assistant observed each child for 60 minutes following ingestion of zinc and recorded regurgitation and/or vomiting events. If an episode of vomiting or regurgitation was observed, the research assistant conducted a brief interview, with 13 questions, the parent gave verbal consent to document illness-history. To compare the illness-history prior to giving zinc, for each child experiencing regurgitation and/or vomiting after the administration of zinc, another child was identified as a control (he/she did not have regurgitation and/or vomiting after giving zinc) by taking the next or the closest child registered. The admission characteristics, preparations, criteria for administration of zinc, and observation period after giving zinc were similar in both the groups. Research assistants similarly conducted brief interview with the parent to document illness characteristics of control patients.

In the SSW, patients were rehydrated using oral or intravenous fluids. Their usual stay in this ward was around 24 hours. Here also, children who met the study criteria received their first dose of zinc when they were considered settled, i.e. no vomiting in the past hour and hydrated. This usually required 6-8 hours. Children then were observed for 60 minutes after the administration of zinc, and all regurgitation and/or vomiting events were recorded. Here also, with verbal consent, the research assistants carried out the interview (13 questions) with the parent. In the similar manner, children for the control group to compare illness were selected, and their illness characteristics were recorded.

While receiving zinc, whether in the SSW or at the PSKP clinic, doctors on duty at these sites assessed all children. In the SSW, the doctors assessed all children at least two times in 24 hours, and the nurses provided 24-hour monitoring of the children for development of any unusual symptoms that might be related to zinc toxicity. Thus, all attending medical staff were advised to report any adverse event that could potentially be attributed to zinc treatment.

Vomiting was defined as the forceful emptying of stomach contents and was recorded if it happened within 60 minutes following the administration of zinc. Regurgitation was defined as the unforceful return of any amount of the swallowed syrup or other stomach contents within five minutes of the administration of zinc.

### Treatment regimen

Every child in the study was prescribed 20 mg of zinc sulphate once daily for 10 days.

### Zinc tablet formulation

Nutriset Ltd. and Rodael Laboratory—both located in France—produced the zinc premix, and a local pharmaceutical laboratory—Square Pharmaceuticals—compressed this into dispersible tablets and packaged the tablets in aluminum blister packs (each tablet contains 20 mg of zinc sulphate). The tablets are vanilla-flavoured, and the metallic taste of zinc is masked using a non-encapsulated, patented technology. The tablets quickly dissolve upon addition of a few drops of water, resulting in a syrup.

### Data analysis

Data were entered and analyzed using the SPSS PC software (version 11.0). Data were re-entered to verify accuracy in a 10% sample of subjects. Errors in data entry occurred in less than 0.5% of the entries. The between-group differences were assessed by chi-square test, and a p value of <0.05 was considered significant. Odds ratios (ORs) and 95% confidence intervals (CIs) were also calculated

## RESULTS

**Fig. 1:**
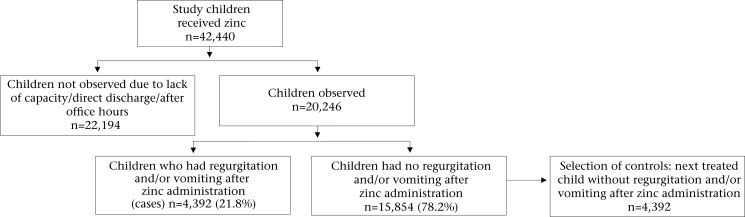
Study profile

During the one-year study period, 42,440 children aged less than five years received zinc, of which 20,246 (47.8%) were observed—12,233 in the PSKP clinic and 8,013 in the SSW. The rest could not be observed either because of lack of capacity at busy times (due to overflow of patients) to observe all children, direct discharge from the triage, and arrival of children before and after office hours and at night when no research assistant was available. Regurgitation and/or vomiting occurred in 4,392 (21.8%) of the children (Fig. 1). Regurgitation alone occurred in 6.3%, vomiting alone occurred in 15.0%, and both occurred in 0.5% of the children. A history of vomiting during the past 24 hours prior to the administration of zinc was present in 85.8% of the children. Of the remaining children, 7.4% had no history of vomiting during their illness, and 6.8% had history of vomiting, but the last episode was before 24 hours of administration of zinc. Vomiting by children after ingestion of zinc occurred once in 90.8%, twice in 8.7%, and more than twice in 0.5% of them.

**Fig. 2:**
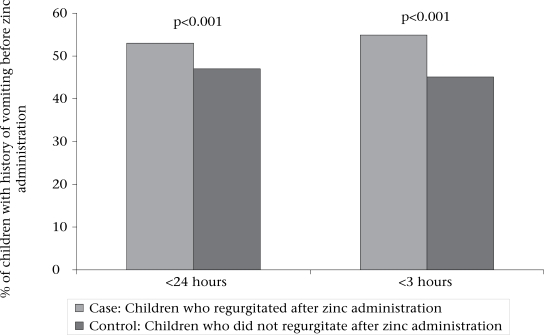
Comparison of illness before administration of zinc

**Fig. 3:**
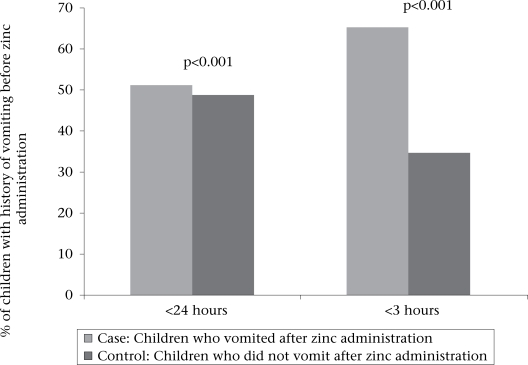
Comparison of illness before administration of zinc

Comparison of illness (before giving zinc) of the two groups showed that significantly more children had a history of vomiting in the past 24 hours among those who regurgitated (53.0% vs 47.0%, OR=2.0, p<0.001)) or vomited (51.2% vs 48.8%, OR=2.2, p<0.001) after ingestion of zinc tablet (Figs. 2 and 3). Similarly, the proportion of children who had a history of vomiting in the past three hours prior to giving zinc was significantly higher among those with regurgitation (54.9% vs 45.1%, OR=2.2, p<0.001) or vomiting (65.3% vs 34.7%, OR=3.9, p<0.001) following the administration of zinc (Figs. 2 and 3). With the exception of vomiting and regurgitation, the attending medical staff did not observe any adverse events that could be attributed to the zinc treatment. No child was brought back to the hospital because of recurrent vomiting after discharge from the hospital.

## DISCUSSION

To our knowledge, this is the first introduction of routine zinc therapy in a large hospital that treats childhood diarrhoea, irrespective of severity, and with or without complications. We used a dispersible tablet containing 20 mg of zinc sulphate. The tablets mask the metallic zinc taste. The advantages of the tablet formulation over the syrup formulation include its much lower cost (about half of that of a syrup formulation), accurate dosing, ease of storage, longer shelf-life, and ease of administration. This study focused exclusively on potential adverse events following the initial treatment with zinc in children aged less than five years, in particular vomiting and regurgitation. In this study, regurgitation and/or vomiting occurred in 21.8% of the children following the administration of the first dose of zinc. This rate of vomiting and regurgitation is very similar to that observed among children receiving zinc in a randomized study conducted concurrently in the same setup by Larson et al. (23). Patient-selection criteria, study preparations, zinc tablet formulation, its dose, and observations before and after giving zinc were same in those children as ours, and they were randomized to one of three groups: no treatment, placebo, or zinc sulphate 20 mg. The study by Larson et al. also showed that 58 (10.9%) of 533 children who did not receive zinc (only ORS) had experienced regurgitation and/or vomiting—regurgitation alone in 2.1% and vomiting alone in 8.8%. The age and sex distribution of those children were comparable with those of our study children. Comparison of the results of these two studies would indicate that the incidence of regurgitation and vomiting occurred in significantly higher proportions of children who received zinc (2 times higher probability) than those who did not receive zinc (21.8% vs 10.9%; OR=2.3, 95% CI 1.72-3.04; p<0.001).

Vomiting following the administration of oral medications is frequently observed among children admitted to our hospital. This background cause of vomiting and vomiting due to diarrhoeal illness cannot be separated from vomiting due to zinc tablet formulation in this study. In this study, more than 90% of the children experienced vomiting only once, and the chances were more for children who had a history of vomiting along with their diarrhoea. This is also consistent with the results of the study carried out by Larson et al. (23). Together, these findings support the early institution of zinc therapy after settling the child during episodes of diarrhoea. There is a risk for vomiting but it occurs only once in the large majority of cases and is unlikely to be of major clinical significance, i.e. interruption of zinc therapy or interference with maintenance of hydration using oral rehydration fluids.

There are important limitations to this study affecting generalization and clinical interpretation of the study's findings. The study population included children who were brought to the ICDDR,B hospital, locally known as the ‘Cholera Hospital’. These children are representative of the more severe end of the illness spectrum. While less than one-half presented with signs of dehydration, over 90% had a history of vomiting during the illness. It appears that vomiting is a major factor affecting the decision to seek help at the Centre. Only 7% of all subjects reported no history of vomiting during their diarrhoeal illness, and 86% reported having vomited in the past 24 hours. The absolute increase in vomiting attributable to zinc will likely be significantly less among children treated at home. A second limitation in this study is the one-hour observation of children. For ethical reasons, it was concluded that children should not be denied zinc therapy, therefore the decision to restrict the period of time for administration of zinc was withheld. This study does not address continued vomiting or vomiting following subsequent doses. A third limitation is the fact that this study used tablets prepared from a single premix shipped from France. It is possible that there are properties unique to this batch that have caused the vomiting.

Future studies monitoring side-effects of zinc need to consider alternative treatment protocols. For example, would an initial dose of 10 mg given twice during the first day lessen the likelihood of vomiting or regurgitation? Longer-term follow-up is indicated addressing such questions as whether or not vomiting continues to occur with subsequent doses and does it continue once the illness subsides. There is also a need to continue to work towards improving zinc tablet formulations that have a minimal risk for side-effects. This could include alternative zinc tablet preparations, such as zinc acetate. The masking of the zinc taste in the tablets is quite effective, but involves a patented technology that does not encapsulate to zinc. This non-encapsulation technique significantly reduces the cost of the formulation, but results in direct exposure of the oesophageal mucosa to zinc that can lead to local irritation and vomiting as a consequence. Encapsulated zinc formulations would be better tolerated, however lower-cost encapsulation techniques need to be developed. Finally, and most importantly, it needs to be confirmed that any additional risk for vomiting or regurgitation does not result in unacceptable levels of clinically significant adverse outcomes.
